# Supplementary figures and images for: Habitat composition near linear landscape structures across Poland: perspectives on pollinator conservation
Source: PeerJ. 2025 Jul 31;13:e19765. doi: 10.7717/peerj.19765 (PMC12318509; doi:10.7717/peerj.19765)

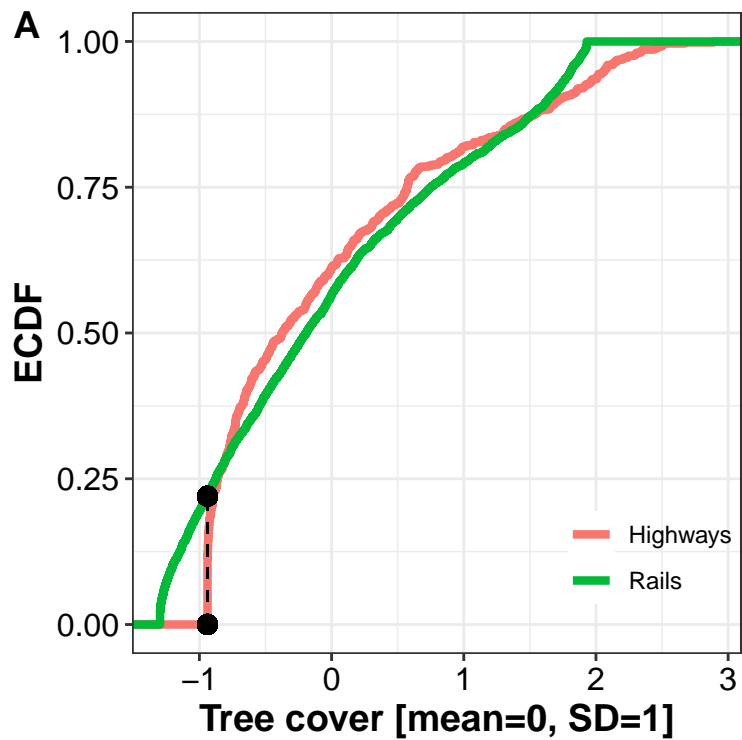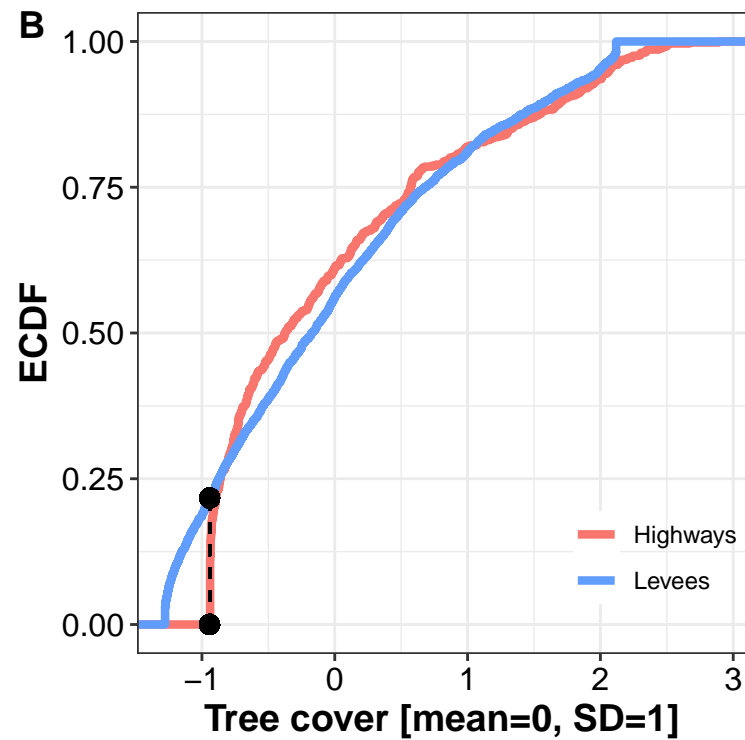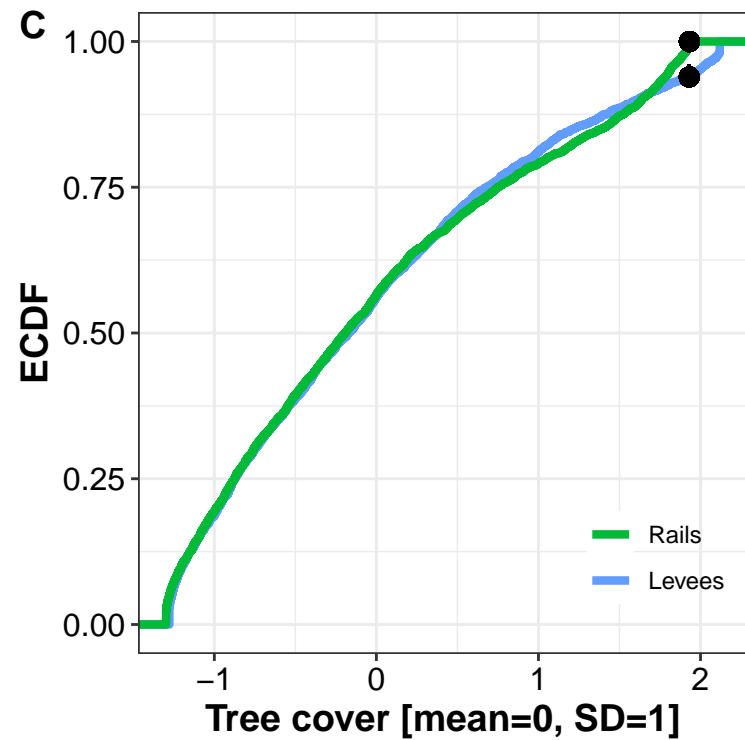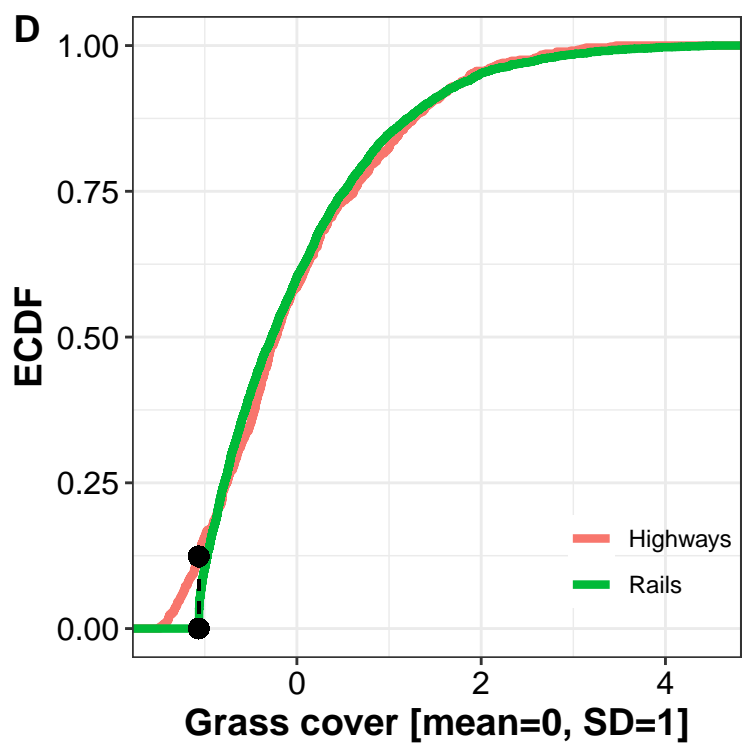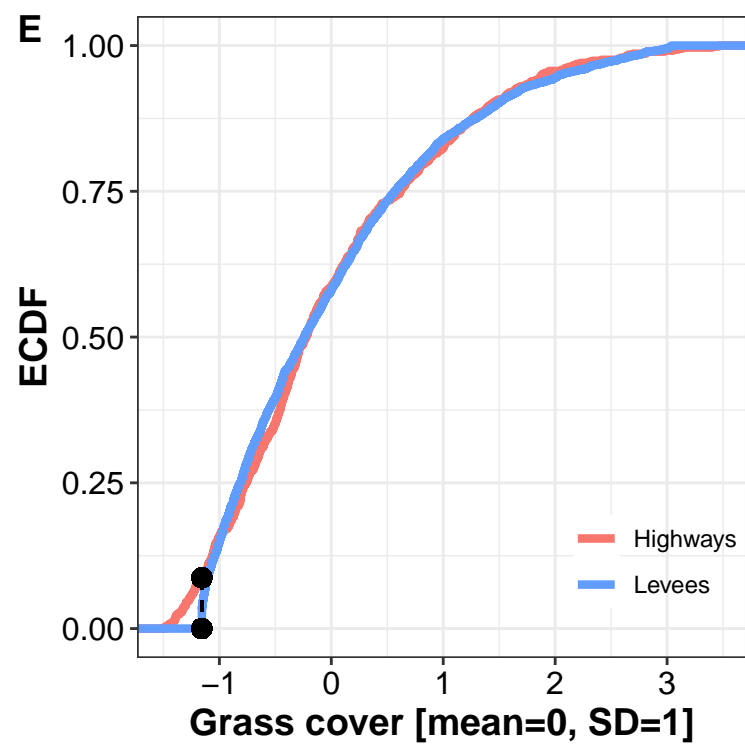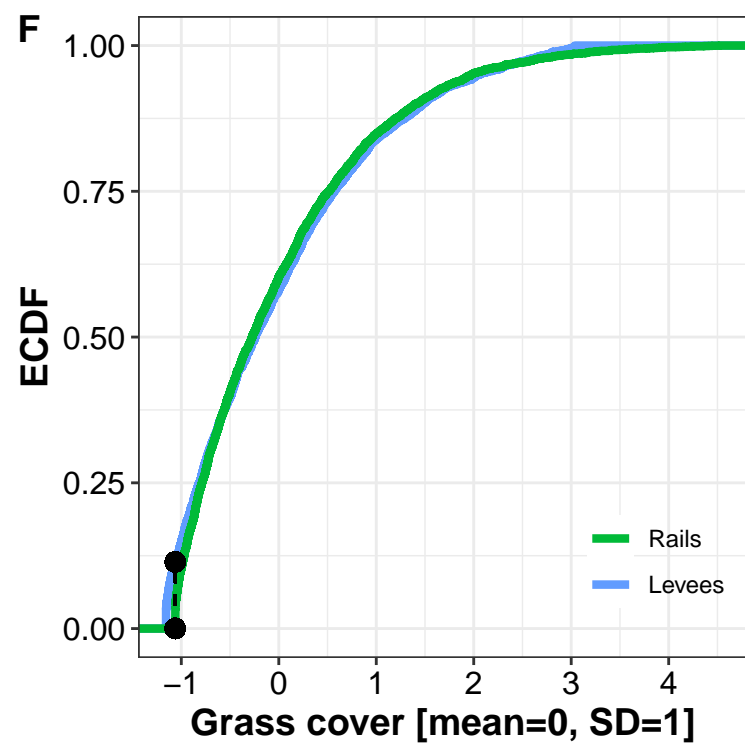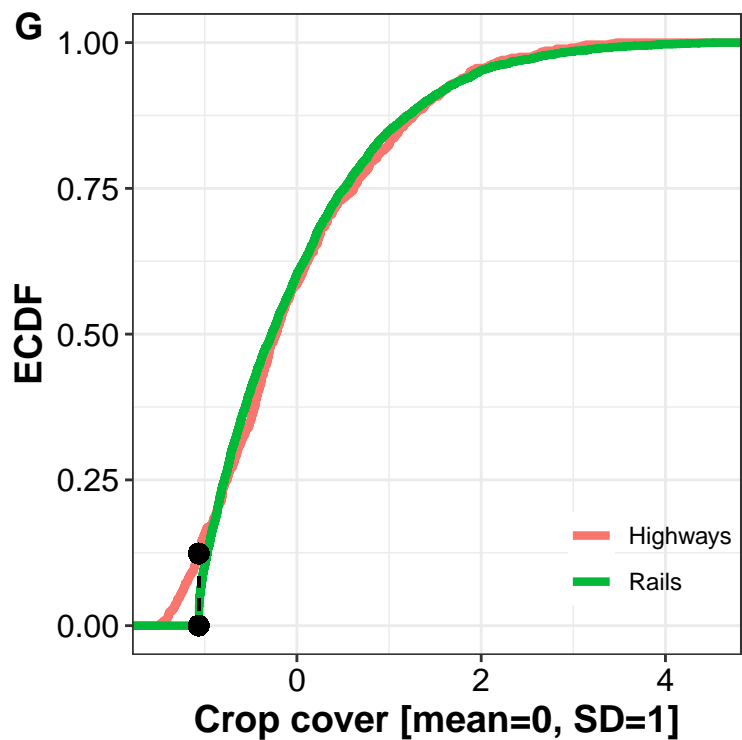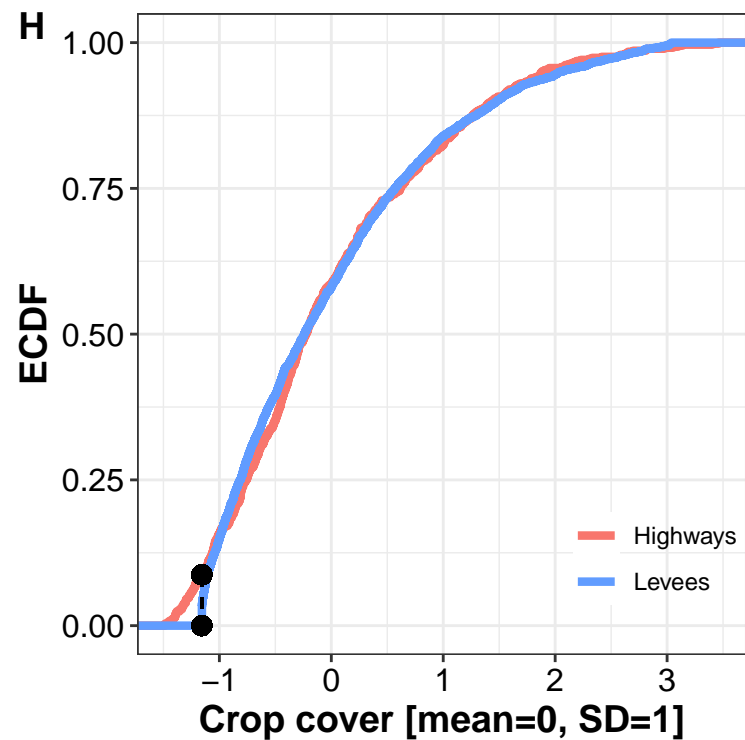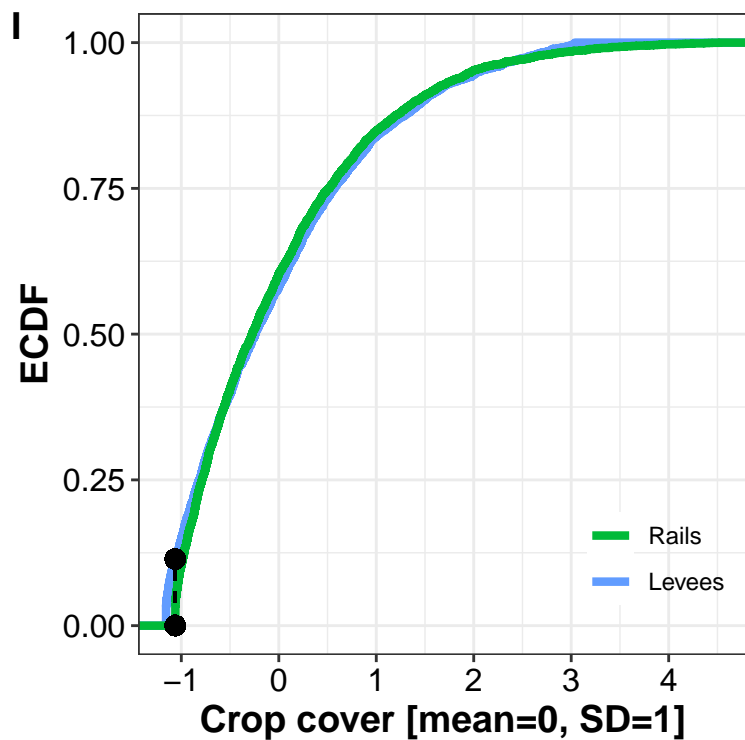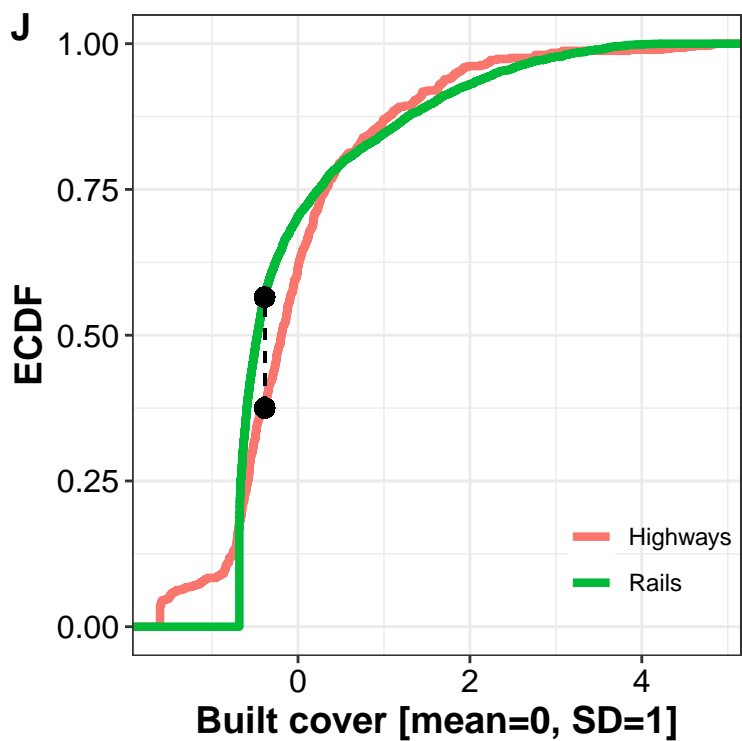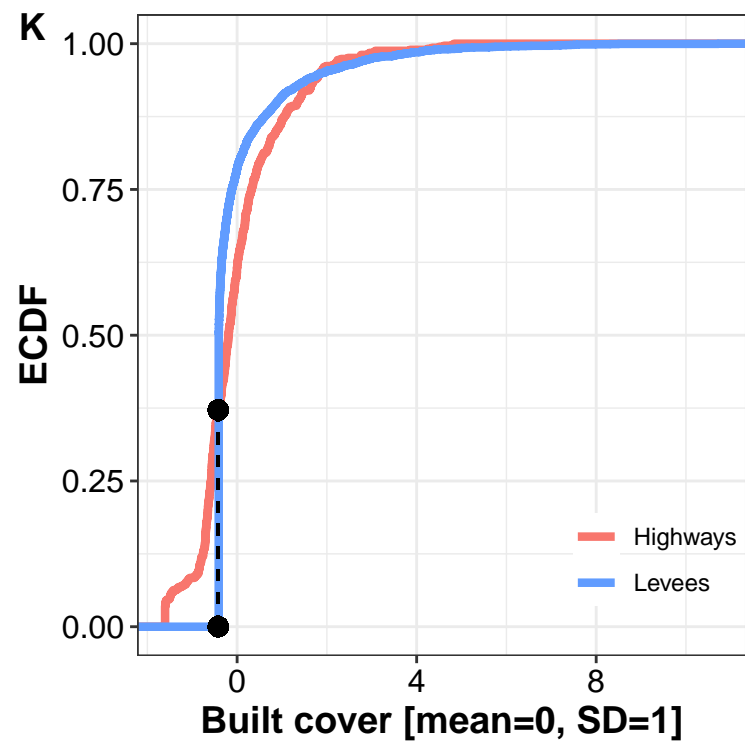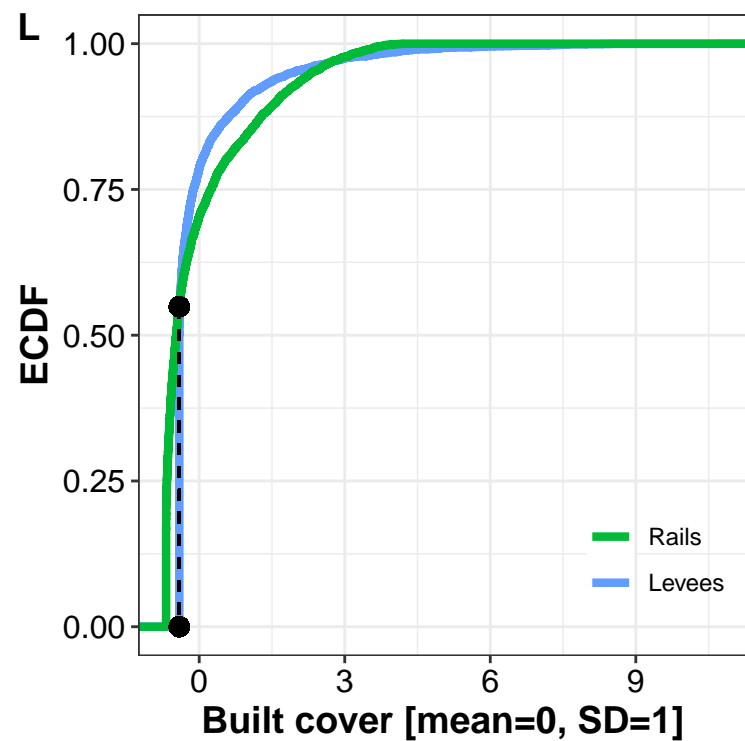

Supplement: Supplemental Information 1 — Differences between empirical distribution functions (ECDF) of linear landscape structures (highways, rails, and levees) landscape covers. Dashed lines between black points indicate the maximum difference between ECDFs. [file peerj-13-19765-s001.pdf]
